# Pathological Researches on Inflammation of the Veins of the Uterus

**Published:** 1830-04-01

**Authors:** 


### MEDICO-CHIRURGICAL TRANSACTIONS.*

PATHOLOGICAL RESEARCHES ON INFLAMMATION OF THE VEINS OF THE UTERUS, WITH ADDITIONAL OBSERVATIONS ON PHLEGMASIA DOLENS. By *Robert Lee*, M.D. Physician-Accoucheur to the British Lying-in Hospital.

WE shall not be at the trouble of informing our readers that this is the age for morbid anatomy, because they must know it as well as we. When Cullen gave his celebrated sketch of the schools of physic, from the days of the old wives who preceded Hippocrates down to those of Sydenham and Boerhaäve; when he portrayed the schisms that have ever raged between the empirics and the dogmatists; and when he framed his own dear nosologic system, little did he dream that a species of knowledge was growing into strength in the dead-house of the hospitals and the studio of the anatomist, which should rival, perhaps overthrow, the theories and the theorists of medicine, and strangle nosology even in its birth. So it seems to be, and the cry of the morbid anatomists and preparation men is—vivent les bouteilles—à bas les nosologies! The whole of the present "part" of the present "volume" of the Medico-chirurgical Transactions is occupied with subjects of morbid anatomy, or something very like it, the first seventy-four pages being a memoir on Adventitious Structures, by our indefatigable friend Dr. Hodgkin; and the last sixty-eight on the Veins of the Uterus, by Dr. Lee. The intermediate matter, consisting of a paper of thirty pages, on Lithotomy, by Dr. Yellowly, completes the "part," which is hardly so weighty, either in bulk or in materials, as some of its predecessors.

The article which we have selected for analysis, Dr. Lee's, is one of much interest in several points of view. Whether we look upon it with reference to venous inflammation generally, to inflammation of the uterine veins in particular,

---

* Vol. XV. Part II.

or to the pathology of phlegmasia dolens, it is calculated to give birth to some reflections in the minds of those who have paid any attention to these subjects. From circumstances, with which we need not trouble our readers, we have seen a good deal of phlebitis, and, consequently, we come to its consideration with a mind more pre-occupied than was a certain President's of the Board of Trade, who announced as his political creed, that his brain was as "a sheet of blank paper." At the same time, we hope we have no material prejudices to clog our sensorial machinery, none, at least, that will obstinately blind us to the perception of truth when she stands before us. So much for ourselves.

Dr. Lee informs us that, in a former communication on Phlegmasia Dolens,* he was led to infer, that inflammation of the iliac and femoral veins "gives rise to all the phenomena of that disease in puerperal women." It will be observed that there is a degree of ambiguity in this passage, for we are left in the dark as to whether such venous inflammation is a cause, amongst others, of the phlegmasia dolens, or whether it is viewed by the Doctor as the whole and sole one. We believe that, taking in the uterine veins, the latter is his opinion; an opinion on which we have joined issue with him before, and will be compelled to join issue with him again. But we anticipate;—let us revert to Dr. Lee. "Subsequent dissections have enabled me," says he, "not only to confirm the accuracy of my former observations, but have led me to discover the important pathological fact, that, in phlegmasia dolens, the inflammation commences in the uterine branches of the hypogastric veins, and subsequently extends from them into the iliac and femoral trunks of the affected side." Here, again, the passage is obscurely or ambiguously worded, for the reader cannot certainly tell, whether the author does or does not affirm inflammation of the uterine veins to exist in all cases of phlegmasia dolens. If the former, we leave him to explain the occurrence of the disease in the male, in whom uterine veins have not hitherto been discovered; if the latter, we must say that the text might have been more explicit. In order to establish the truth of views thus imperfectly explained, Dr. Lee subjoins a number of cases, of which we shall select and condense as many as our limits will enable us to comprise within this article.

Case 1.—*Inflammation of the principal Abdominal Veins and those of the right Inferior Extremity.* Mrs. Edwards, æt. 35, was delivered of her second child after a natural labour, and a fortnight afterwards, viz. April 9th, 1829, was attacked with pain in the calf of the right leg, and loss of power of that lower extremity. On the 13th, the limb was swollen without discoloration, and the inner surface of the thigh to the groin was very tender upon pressure. On the 16th the swelling was universal, the integuments pale and glistening, and not pitting upon pressure, great tenderness along the course of the crural vessels, and the vein, from the groin to the middle of the thigh, indurated, enlarged, and exquisitely sensible. There was also great sensibility in the ham, and along the inner surface of the leg to the ankle, where some branches of the superficial veins were hard and painful upon pressure. There was little pyrexia, had been no rigor, and she said that the veins of this extremity had been the most distended during pregnancy. Twelve years previously, after the birth of her first child, she had experienced a similar attack in the same limb, which remained in a weak condition for several months.

The affection of the thigh diminished after the lapse of a week or ten days, but she became affected with rigors, quick pulse, &c. and complained of considerable uneasiness between the umbilicus and pubes and in the loins. The rigors came on every afternoon and were followed by heat and perspiration, the attacks of pain were acute, there was slight delirium at night, the fever was typhoid, there was soreness around the umbilicus, and pulsation in the epigastrium. These symptoms declined, but on the 20th of May she had another violent rigor,

* Page 132 of the present volume.

vomiting succeeded, and pain in the left side on deep inspiration; then appeared great prostration of strength, a peculiar sallowness of the skin, inflammation of the right eye, and delirium at night. The left eye also inflamed, the prostration increased, rigors took place from time to time, with hacking cough, diarrhœa, more or less insensibility, and the usual symptoms of typhus. On the 31st of May the eyes were so much swollen that they seemed pushed out of their sockets, and vision was entirely lost. On the 2d of June, a red puffy swelling appeared over the right elbow-joint, and on the 15th she died.

*Sectio Cadaveris.* Present, Drs. Sims and Locock.

"*Thorax.*—In its left cavity were contained upwards of two pints of a thin purulent fluid, and extensive recent adhesions existed between the pleura covering the lower margin of the superior lobe and the pleura costalis. The surface of the inferior lobe was coated with a thick layer of flocculent coagulated lymph, as was a corresponding part of the pleura costalis. The substance of this lobe was of a dark colour, approaching to black, and soft in texture, so as to be readily broken down with the fingers. In its centre about an ounce of thick cream-coloured pus was found deposited in the dark-coloured and softened lung. This was not contained in any cyst or membrane, but infiltrated into the pulmonary tissue.

"In the right cavity of the chest recent adhesions also existed at the inferior part. A considerable portion of the right inferior lobe was entirely changed from the healthy structure, being converted into a dense, solid, dark red coloured mass. On the anterior surface of this lobe the pleura was elevated as if by a hard irregular tumour, but when cut into, no pus escaped from this part, and it presented only the appearance of the surrounding portions of lung with a greater degree of condensation.

"*Vena cava inferior.* Coats of the vessel considerably thickened, and the internal, where visible, of a scarlet colour; its whole cavity occupied by a coagulum, distending it to its utmost extent, and terminating in a loose pointed extremity about an inch below the entrance of the vena cava hepatica. The coagulum, covered with a membranous-like investiture of a bright red colour, throughout firmly, and in many places inseparably adherent to the inner lining of the vein; the substance within it varied in consistence and colour, in some parts it presented the appearance of coagulable lymph, in others it was a pultaceous dull yellow mass, made up apparently of pus and lymph blended together. The exterior of the firmer portions were separated into layers, which gradually disappeared as they approached the centre. The mouths of all the veins emptying themselves into the cava were sealed up, the emulgents excepted, the coagulum, near the entrance of these vessels, hanging loosely within the cava.

"*Left common iliac and its branches.* Its interior plugged up with a continuation of the coagulum from the cava, and differing in no respect from it either as to consistence, colour, or the firmness of its adhesions to the inner tunic of the vein; it was continued beyond the entrance of the internal iliac, (which it completely closed,) and terminated in a pointed extremity about the middle of the external iliac; neither the remainder of this vessel nor the femoral vein exhibited any morbid changes. The internal iliac was much contracted and lined with a thick adventitious membrane.

"*Right common iliac and its branches.* This vessel was contracted to more than one half its natural size; it was firm to the touch, and of a grayish blue colour, to its internal coat adhered an adventitious membrane of the same colour, containing within it a firm coagulum, made up of thin layers of dense lymph. The internal iliac was rendered quite impervious by dense dark-coloured bluish membranes, and at its entrance into the common iliac was converted into a solid cord.

"The contracted external iliac contained within it a soft yellowish coagulum, similar to the one in the cava; its coats were three or four times their natural thickness, and lined with dark-coloured membranous layers.

"The *femoral vein*, from Poupart's ligament to the middle of the thigh, was diminished in size, and almost inseparable from the artery. Its tunics were thickened, and its interior coated with a dense membrane surrounding a solid purple coagulum strongly adherent to it. The superficial and deep femoral veins were in a similar condition, and the saphena major and minor differed from the femoral veins only in the size of the coagulum they contained, which was slender, and had formed no adhesions with the layers of lymph lining their cavity.

"The cellular membrane and other textures of the limb were in a perfectly healthy condition, and in size and appearance there was externally no visible difference between the two extremities.

"The morbid alterations of structure now described, can still be distinctly seen in the preparation of the diseased veins, and have been represented with great accuracy in the beautiful drawing made by Mr. Perry, from the parts immediately after their removal from the body." 378.

In the second case the patient was confined in the latter part of March, whilst labouring under phthisical symptoms, and on the 4th of May experienced soreness in the left groin which gradually extended along the inner surface of the thigh to the ham, and thence along the back of the leg to the foot. In twenty-four hours the limb began to enlarge, and the swelling became hot, painful, and colourless, pitting nowhere on pressure, except over the foot. Motion produced excruciating pain along the inner surface of the thigh, and the pain along the tract of the femoral vein was so acute that the condition of this vessel could not be ascertained. Several branches of the saphena above the knee were distended and hard; pulse 120; tongue red and glossy. On the 11th the femoral vein under Poupart's ligament could be felt enlarged and indurated, on the 17th there was less pain at the groin and in the course of the vessels, the pulmonary affection became aggravated, and in the morning of the 24th she died.

*Sectio Cadaveris*:—Vomicæ, &c. in the lungs. Left common, external, and internal iliac veins all impervious, with various alterations of structure. Common iliac at its termination reduced to a very slender tube, lined with a bluish slate-coloured adventitious membrane. Remainder of the common and external iliac veins coated with a dark-coloured membrane, and their centre filled with a brownish ochrey-coloured tenacious substance, rather more consistent than the crassamentum of the blood.

Left internal iliac vein in some places reduced to a cord-like substance, and its cavity throughout completely obliterated; its uterine branches completely plugged up with firm reddish coagula of lymph. Branches and trunk of right hypogastric vein affected in same way as the left. Coats of left femoral vein thickened, and closely adherent to the artery and surrounding cellular substance; its whole interior lined with an adventitious membrane, and distended with a reddish coloured coagulum. Same morbid appearances in deep and superficial branches as far as examined down the thigh.

Case 3.—*Phlegmasia Dolens—Iliac and Femoral Veins inflamed.* Mrs. Mason, æt. 42, was delivered Aug. 1, 1829, of twins, and before the expulsion of the placenta had nearly perished of uterine hæmorrhage. Much tenderness of the uterus remained till the 27th, when she had a violent rigor, succeeded by fever, and pain in the right iliac region and groin. On the 28th the pain increased and extended towards the ham, and in the evening the limb swelled. On the 29th, the femoral vein for several inches under Poupart's ligament was felt enlarged and painful; tenderness in the right side of the hypogastrium; deep seated acute pain in the lower part of the spine on motion; depression. Sept. 8. Less pain in limb—femoral vein still enlarged and painful—foot and leg pit—rigors and occasional attacks of diarrhœa. These symptoms continued with little variation except that she complained of tenderness in the left groin and thigh, and was at times delirious. Before her death, which happened on the 22nd, both inferior extremities were œdematous.

*Sectio Cadaveris*:—The veins presented nearly similar appearances to those observed in the preceding cases. On the right side the iliacs were affected and imbedded in a mass of suppurating glands and pus which extended in the cellular membrane along the psoas muscle to Poupart's ligament. Lower two inches of the vena cava affected like the iliacs. On the left side the common, external iliac, and hypogastric veins contained soft adherent coagula, and the latter vessel was somewhat contracted and thickened in its coats.

The three succeeding cases were not fatal, and consequently no opportunity was afforded of establishing in a positive manner the existence of phlebitis. In all, however, there was pain and induration in the course of the femoral vessels, much swelling of the thigh or of the limb and tenderness to touch, slight pitting upon pressure, and more or less constitutional disturbance and depression. In the second, or fifth, case, the inguinal glands were enlarged and suppurated.

Some remarks are made on the preceding cases by Dr. Lee with the view of shewing that phlebitis did actually exist, and that it originated and "generally commences" in the branches of the hypogastric, or uterine veins. The first of these conclusions will admit of no dispute, and need not be dwelt on further; the latter is rendered probable by the cases brought forward by the Doctor, as well as by the mention of others which he cites from various authors. Mr. Wilson in the Transactions of a Society for the Improvement of Medical and Chirurgical Knowledge, Vol. II. relates three cases of inflammation more or less extensive of the vena cava, iliacs, and uterine veins, in none of which was there *one of the symptoms of phlegmasia dolens*. M. M. Meckel, Bouillaud, Lawrence, Velpeau, and Guthrie, with Drs. Davis and Gooch, are quoted in support of the position in question, some of them satisfactorily, others not. This marshalling of cases and array of authorities, some of whom may be said to be rather *pressed* than fairly enlisted, is terminated by Dr. Lee asserting that "phlegmasia dolens must now be considered as merely one of the remote consequences of uterine phlebitis."

Dr. Lee proceeds to trace the mode in which uterine phlebitis is set up, its progress, symptoms and sequelæ. Generally the "spermatic veins alone are affected, and for the most part only that on the side of the uterus to which the placenta has been attached; and inflammation being once induced it is liable to spread continuously to the veins of the whole uterine system, of the ovaria, fallopian tubes, and broad ligaments. The vena cava itself may become affected, but this occurrence is not frequent, the disease being usually arrested at the entrance of the spermatic into the vena cava on the right side, and of the emulgent into the same vessel on the left. If, as sometimes happens, it pursue the direction of the kidneys, the substance of these organs, as well as their veins, may be involved in the mischief. The hypogastric veins are seldom affected on both sides, and rarely inflamed in comparison with the spermatic.

"Uterine phlebitis appears to result from the mechanical injury inflicted by protracted labour, from the force required for the extraction of the placenta in uterine hemorrhage, from retained portions of placenta undergoing decomposition in the uterus, the application of cold and probably of contagion, and from various unknown causes operating on the uterine system after delivery.

"It is perhaps impossible to determine for the most part, the precise period of its invasion, from the total absence of local pain, and of other symptoms; but it is probable that it most frequently begins soon after delivery, and remains stationary for a time around the orifices of the uterine veins, as phlebitis has been observed to do, where it occurs after venesection. Of this, however, we can have no certain proof, nor can it be admitted to be a general occurrence, from the rapidity with which the inflammation has been found to attack the uterine, spermatic, and renal veins. In one case the disease proved fatal on the evening of the fifth day after labour, and on dissection, all these veins were found disorganized.

"It may be stated, as the general result of all the observations hitherto made on uterine phlebitis, that it occurs most frequently from the 10th to the 20th day after parturition, though it has been observed to commence at an earlier, as well as at a much later period.

"Where the veins alone are inflamed, the peritoneal and muscular tissues remaining unaffected, there is often either no pain or only a dull pain, with a sense of weight in the region of the uterus, and no other local symptom by which the disease can be recognized. The uterus too may return to its usual reduced volume or nearly so, and it is only on the accession of the constitutional symptoms, which have been already detailed, that the existence of this insidious and dangerous affection can be determined. If the substance of the uterus be affected, this organ remains above the brim of the pelvis, large, hard, and painful on pressure, as in puerperal peritonitis.

"With regard to the lochial discharge, it has sometimes been observed to be fœtid, and puriform, and at other times in a perfectly natural state." 404.

The constitutional symptoms of uterine phlebitis are like those of phlebitis elsewhere, and need not be enumerated after what has been said in recent numbers of this Journal. Dr. Lee is induced to believe that the disease is of much more frequent occurrence than has hitherto been suspected, and "that to it must be referred many of the fatal disorders of puerperal women which have usually been comprehended under the vague designation of puerperal fever or peritonitis." According as the serous, muscular, or venous tissue of the uterus may be affected, will the forms of puerperal disease, in Dr. Lee's opinion, be inflammatory, congestive, or typhoid.

A kind of halting-place now occurs in the paper and we cannot have a better opportunity than this of making the few remarks which we intend to offer on the subject. Our readers are probably aware that we have on several occasions dissented from the pathology of phlegmasia dolens first promulgated by Dr. Davis, subsequently supported, and now extended by Dr. Lee. Our opposition neither originated in a spirit of opposition, nor was continued in one of obstinate pertinacity. We believed, in the first instance, that Dr. Davis had not proved his case, and though our opinion may be modified, it certainly is not subverted by what has since occurred. Not to go over the grounds of our opposition to the theory which places the exclusive origin of phlegmasia dolens in inflammation of the veins, we may here allude to some circumstances deserving of consideration.

It cannot fail to be remarked that a considerable discrepancy exists between the disease as described by the older writers, and many of the cases detailed in these modern papers. Mr. White, who wrote in 1784, mentions that out of 1897 women delivered at the Westminster General Dispensary, five only were attacked with phlegmasia dolens; and out of 8000 delivered at the Manchester Lying-in-Hospital, and their own houses, only four;—whereas our modern practitioners would appear to find them as plenty as blackberries. Again, the phlegmasia dolens of the old schoool is by no means a fatal disease, on the contrary Mr. White declares that, when not complicated with any other disease, he has never known it have a fatal termination. Dr. Hull, to be sure, informs us that he has seen cases end in suppuration, and even in death, but he speaks of these as somewhat rare occurrences. Now the phlegmasia dolens of the new school is one of very great danger indeed, attended with grave and typhoid symptoms, and followed by sequelæ never alluded to before the present day.

But this is not all. Not only are these great discrepancies between the disease as described of old and of latter years, but even amongst the several cases of our modern phlegmasia dolens. If the old descriptions are to be taken as a standard of the disease, then some of the cases related by Dr. Lee and others must certainly be rejected. Take for instance the first case on the list, that of Mrs. Edwards; it is an excellent specimen of the consequences, immediate and remote, of inflammation of the veins, but none of phlegmasia dolens. The same may be said of some others, more particularly of the three cases quoted from the paper of Mr. Wilson, in which there was plenty of phlebitis, but not a morsel of phlegmasia dolens. Dr. Lee endeavours to get out of this scrape by supposing that the inflammation of the hypogastric vein *only* produces the disease *when* it has extended into the principal veins of the extremity. The reasoning is hardly logical, indeed may well admit of the reductio ad absurdum were any one inclined to apply it; but it is upset by the fact of phlegmasia dolens occurring in the male, in whom it had no uterine veins to arise in. But allowing, as we are really inclined to do, that inflammation of the iliac and femoral veins in the female after parturition, does commonly originate in the uterine branches, still if it can be proved that the swelling of the limb is only set up when the latter is established, the two must be connected as cause and effect, by all the rules of right reasoning and the dictates of common sense.

When we look at the instances of phlebitis which the practice of surgery affords, we are equally at a loss to discover a state of things corresponding to phlegmasia dolens. In inflammation of the veins of the arm after venesection, it is true that swelling of the limb takes place, but there is generally much disposition to œdema, and more or less discolouration of the integuments. In inflammation of the saphenæ or femoral veins, the same is frequently observed, and even when the skin remains white, there is more œdema than in the old, and we may say orthodox phlegmasia dolens. Nay in some cases of inflammation of the iliac and femoral veins which we have witnessed there was little or no perceptible swelling of the limb at all, and none of that exquisite pain on pressure which characterizes the affection under consideration.

*Case.* A young man, 22 years of age, was admitted into St. George's Hospital under the care of Mr. Hawkins on the 15th of November, 1829, with severe gun-shot wound of the right arm and elbow-joint. He refused to submit to amputation, and attempts were made to save the limb, but swelling, erysipelatous inflammation, and sloughing of the cellular membrane of the limb succeeded, with diarrhœa, prostration, cough, and muco-purulent expectoration. From this state he rallied, whilst a wasting suppuration was set up in the limb, and the night-perspirations with the other symptoms of hectic were profuse. On the 21st of December the limb was amputated. For a few days he appeared to be doing well, but on the 26th it was necessary to open a large abscess over the sacrum, when half a pint of fœtid pus mixed with blood was evacuated. The night-sweats persisted, on the 1st of January he had a long-continued rigor, the soft parts of the stump retreated from the bone, and on the 4th the poor fellow died.

*Sectio Cadaveris.* Adhesions of pleuræ on left side of chest—consolidation of lungs, not peripneumonic—hepatization of the lungs in parts—a small abscess, the size of a pea, on the surface of the right lung—no tubercles.

Large sloughy abscess over the sacrum, with portions of dead bone exposed, and a great quantity of coagulated blood. Another large abscess in the left side of the pelvis, extending from Poupart's ligament to the sacro-sciatic notch, but not communicating with the former abscess. External iliac and femoral veins in the neighbourhood filled with half-organized, laminated coagulum, semipurulent in its centre.

An abscess in the calf of each leg.

In this case there was no perceptible swelling of the limb whatever, nor had the slightest pain in it been complained of during life. In another case of inflammation and obstruction of the femoral vein, which we witnessed some time ago at the same hospital, there was tenderness in the course of the vessels, but little swelling, and that not resembling, in the slightest degree, the phlegmasia dolens. The case was that of a man named Frederick Wells, whose leg was amputated by the late Mr. Rose, for compound fracture of the ankle-joint; it was published in the Medical Gazette, and has been copied into Mr. Arnott's memoir on Phlebitis. In a man whose thigh was amputated last Summer by Mr. Keate, and in whom the femoral and iliac veins inflamed, there was not even pain on pressure, and no swelling of any consequence. We might mention some other examples of precisely the same description, but it would merely be exhausting the patience of our readers to no purpose.

From the arguments we have adduced, the facts we have observed, and some other considerations, into which we cannot enter at present, we are led to doubt and dispute the correctness of Dr. Lee's position, that phlegmasia dolens is now to be considered as merely one of the remote consequences of uterine phlebitis. But while we think that Dr. Lee, and the other medical men who have laboured in the same vineyard with him, have failed in establishing the soundness of their opinion as an axiom in pathology, we are willing to admit, and ready to avow, that they have effected a great deal in this department of medical science. They have proved that many cases, so closely resembling phlegmasia dolens as hardly to admit of being distinguished from it, are essentially inflammation of the iliac or femoral veins; and they have rendered it probable, that the majority of the instances of that affection are dependent immediately or remotely on venous inflammation. We cheerfully make this acknowledgment, which is due to the perseverance and laborious research of Dr. Lee and his confrères.

Farther than this we cannot go, nor, in the present state of our knowledge, do we think that it would be right to do so. It is much more reasonable and philosophic to advance cautiously, than rush on with precipitate rashness; in the first case the progress is sure, although it may be slow—in the second we may fall into fatal dangers; and even if we escape, it is only at the expense of toilsome retreats, and many a bewilderment amidst the treacherous mazes of error.

We believe that many of the disputes on the nature of phlegmasia dolens are like the celebrated rencontre between the knights on the different sides of the shield, or the quarrel in the fable on the colour of the chameleon. It seems to us that the enlargements of the extremities in parturient women, and in patients, male and female, not parturient, are of many and various kinds. Surely nothing can present a greater contrast than an ordinary case of phlegmasia dolens, and one of those frightful cases of venous inflammation described in the present paper and in others, in which purulent depositions take place in various parts of the body, in the joints, in the cellular membrane; with sloughing of the eyes, gangrene and sloughing of the lungs, the worst description of typhus, and so on. Surely such cases must differ in something more than in degree; surely it is natural to conclude that the lower extremities are subject to several various affections, of very opposite degrees of suffering and danger. Take, for instance, the following case, related by Dr. Lee, of "severe affection of the joints after parturition."

*Case.* "Mrs. Pope, æt. 40, No. 7, Feathers Court, Drury Lane. She was delivered on the 26th Oct. 1827, of her fourteenth child after an easy labour, and appeared to recover favourably until the 3d of November. Without any obvious cause, she was then suddenly attacked with a severe rigor, which was speedily followed by intense headache, vomiting, general soreness of abdomen, and suppression of the lochia.

"Nov. 6th, 1827, (eleventh day after parturition). The symptoms now observed are, great prostration of strength, laborious respiration, with pain at the bottom of the sternum, and frequent hacking cough: pulse 135, and extremely feeble; skin hot and dry; the lips parched; and teeth covered with brown sordes; tongue of a deep red at the edges, dry, chapped, and covered with a yellow fur in the centre. Occasional retching and vomiting; bowels confined; lochia suppressed. The abdomen is perfectly soft and natural, but feels generally sore on being pressed. She complains of acute lancinating pain in the vertex, and of pain and loss of power to move the left inferior extremity.

"On examining the limb, there are several hard lumpy cords found running up on the inside of the thigh, in the direction of the superficial veins, which are very painful to the touch. The integuments over these are not discoloured.

"The middle finger of the left hand is also exquisitely painful, and on examination, is perceived to be much swollen around the second joint, where the integuments are of a dusky red colour.

"7th. She has been delirious in the night, and is now incoherent, with a peculiar wildness of expression in the countenance. The general debility has greatly increased; the respiration is still more hurried; and the pulse is 140, soft and compressible; the tongue is brown and dry; the muscles of the face and extremities are affected with tremors; the whole surface of the body is covered with a yellow suffusion.

"8th. She is in all respects worse; there has been violent delirium during the night: and she is now roused with difficulty. The respiration is still more oppressed, and the pulse so rapid and feeble as not to be counted. The countenance dejected and deeply suffused, as is the whole surface of the body. The swelling in the joint of the finger has increased, and another painful diffused swelling along the fore-arm has occurred in the night, with slight discoloration. The whole of the right superior extremity has also become stiff, and so painful, that attempts to move it produce violent pain. The swelling and hardness in the course of the superficial veins of the thigh are diminished.

"9th. Complete collapse took place, and she sunk in the course of the afternoon. On the 10th I opened the body, with Mr. Prout of Welbeck-street, who occasionally saw her with me during the progress of the disease.

"*Dissection.*—The intestines were distended with gas; their peritoneal coat had every where a healthy appearance, except a small portion covering the ileum, which was of a bright red colour, though it was not sensibly thickened. The lower part of the omentum, and portions of the mesentery and mesocolon, were also more vascular than usual, but no lymph was effused in these situations. The mucous membrane of the stomach, small and great intestines, was remarkably pale and bloodless. The left Fallopian tube, and fundus of the uterus, was of a deep red colour, but the sinuses of the uterus, and its muscular coat, were quite healthy. Permission was not obtained to examine the head, chest, or extremities." 423.

In another case related by Dr. Lee, the patient had suffered, during the latter months of gestation, from œdema and a varicose state of the veins of the lower extremities. Two days after her confinement she began to complain of pain in the superficial veins of both legs, and, during the subsequent week, a diffuse swelling and erysipelatous redness of the surface took place in the calf of the left leg, and, to a less extent, in that of the right. The usual constitutional disturbance from phlebitis ensued, and on the seventh day, the veins being laid open in two places, a considerable quantity of purulent fluid was discharged. Two abscesses formed above the left ankle and were opened, and a small abscess also formed above the right knee. The patient sank, and died on the 14th day from the commencement of the symptoms.

*On dissection*:—The cellular membrane of the extremity swollen and infiltrated with red-coloured serous fluid—several abscesses beneath the skin in the calf of the leg, and an extensive collection of purulent fluid in the interstices of the gastrocnemii muscles. The branches of the saphena converted into impervious cords, the saphena itself lined with adventitious membrane, the coats of the femoral vein, between the opening of the saphena and the ham, thickened and corrugated, the femoral, above the junction of the saphena, and the external iliac, thickened, contracted in diameter, and lined with a thin coating of lymph.

Here there was inflammation of the veins of the extremity in abundance, but no phlegmasia dolens. Our author likewise gives the case of a lady who had suffered for some time from cancer of the os uteri, and was suddenly seized with vomiting, diarrhœa, and severe pain of the uterus. She lived from the 9th of May, when she was attacked, to the latter end of June, when she died, and was examined by Mr. Griffith, of Tottenham Court Road. *On dissection*, the uterus was found partly destroyed by cancerous ulceration, and the uterine branches of the left hypogastric vein, the left spermatic, and the veins running along the side of the body of the uterus were found more or less plugged up and lined with lymph.

Some other cases of a similar kind are related by our author, and the paper terminates with an appendix, consisting of a case from Mr. Cæsar Hawkins, and one from Mr. Copland Hutchison. The former is curious, from the absorbents and receptaculum chyli, not the veins, being filled with pus. The patient had been brought to bed in St. George's Hospital, and was attacked, two days after delivery, with symptoms of puerperal peritonitis of a low character, of which she died in two days more.

Here our analysis of Dr. Lee's memoir terminates, and comment would be superfluous after what we have already said. We regret that our narrow limits have prevented us from indulging in some remarks that we could have wished to offer, and prevented our entering on some questions that we could have wished to discuss. Regrets, however, on this occasion are unavailing, and a future opportunity may perhaps leave us nothing to deplore. We can only say that we have derived much instruction from the perusal of Dr. Lee's memoir, and we recommend every member of the profession, whether engaged in the accoucheur department or not, to weigh attentively the valuable facts it contains. We part from the Doctor with many thanks.

